# Aberrant Emotion-Cognition Integration in *FMR1* Premutation Carriers: A fNIRS Study

**DOI:** 10.1523/ENEURO.0031-26.2026

**Published:** 2026-08-12

**Authors:** Yuewen Bian, Yuhang Li, Yiting Zhu, Dongyun Li, Yingbo Geng, Shuo Guan, Danyong Feng, Rihui Li, Qiong Xu

**Affiliations:** ^1^Centre for Cognitive and Brain Sciences, Institute of Collaborative Innovation, University of Macau, Macau S.A.R. 999078, China; ^2^Department of Psychology, Faculty of Social Science, University of Macau, Taipa, Macau S.A.R. 999078, China; ^3^Department of Child Health Care, Children’s Hospital of Fudan University, Shanghai 200032, China; ^4^Department of Electrical and Computer Engineering, Faculty of Sciences and Technology, University of Macau, Macau S.A.R. 999078, China

**Keywords:** *FMR1* premutation carriers, fNIRS, Go/No-Go task, PMC, Stroop task

## Abstract

Carriers of the *FMR1* (Fragile X Messenger Ribonucleoprotein 1) premutation (55–200 CGG repeats on the X chromosome), known as premutation carriers (PMCs), often exhibit executive dysfunction. Yet, the neural mechanism of cognitive and executive dysfunction in PMCs remains underexplored. This study investigated the neural alterations associated with the inhibitory control and conflict processing of PMCs using functional near-infrared spectroscopy (fNIRS) technique. We recruited 31 participants, including 14 participants with the *FMR1* premutation (all females, mean age: 36.93 ± 8.06 years) and 17 controls (3 male and 14 females, mean age, 30.86 ± 5.90 years). Hemodynamic activity of the participants during emotional Go/No-Go and Stroop tasks was recorded using a fNIRS system. The neural activation in response to different tasks was examined. Compared with the control group, the PMCs group exhibited decreased activation in the left dorsolateral prefrontal cortex (DLPFC, *p* = 0.03) during the Stroop task. They also revealed increased activation in the right DLPFC (*p* = 0.015), right frontopolar cortex (FPC, *p* = 0.027), right Broca's area (*p* = 0.013), left premotor and supplementary motor area (SMA, *p* = 0.013), and visual association cortex (*p* = 0.044) during the Go/No-Go task. Conversely, the PMCs group exhibited decreased activation in the right premotor and SMA (*p* = 0.018) and the right somatosensory association cortex (*p* = 0.013). Our findings revealed atypical neural patterns in response to the Stroop and Go/No-Go tasks, suggesting the underlying neurobiological mechanisms of inhibitory control and conflict processing are disrupted in PMCs, potentially guiding targeted interventions.

## Significance Statement

This study investigated the neural mechanisms underlying executive dysfunction, specifically inhibitory control and conflict processing, in *FMR1* premutation carriers using fNIRS. The findings revealed atypical neural activation patterns in premutation carriers compared with controls. During the Stroop task, the premutation carrier group showed decreased activation in the left dorsolateral prefrontal cortex. During the Go/No-Go task, they exhibited a mixed pattern, including increased activation in right-hemisphere regions and decreased activation in the right premotor and supplementary motor area. This study identifies distinct neural patterns associated with inhibitory control and conflict processing in premutation carriers, suggesting that the underlying neurobiological mechanisms governing these processes are disrupted, which could potentially guide future targeted interventions.

## Introduction

Approximately 1 in 113–178 females are carriers of the Fragile X Messenger Ribonucleoprotein 1 (*FMR1*) premutation, which is caused by an expansion of 55–200 CGG repeats in the *FMR1* gene on the X chromosome (Hantash et al., 2011; Seltzer et al., 2012; Moser et al., 2021). In individuals with the *FMR1* premutation, transcriptional upregulation results in elevated levels of *FMR1* mRNA (Seltzer et al., 2012; Moser et al., 2021; Hessl et al., 2024). The excess mRNA, particularly the expanded CGG repeat-containing transcripts, is believed to induce cellular dysfunction through a mechanism known as RNA toxicity, contributing to a range of clinical phenotypes (Tassone et al., 2000; Kenneson et al., 2001). Increasing evidence shows that premutation carriers (PMCs), especially females, are at heightened risk for mood disorders such as anxiety and depression (Bourgeois et al., 2007; Hessl et al., 2011; Shelton et al., 2016). While Fragile X-associated tremor/ataxia syndrome (FXTAS) and primary ovarian insufficiency (FXPOI) represent well-recognized clinical outcomes of the premutation, mood-related disruptions are now acknowledged as another critical clinical outcome. These emotional and neuropsychiatric manifestations are formally encompassed under Fragile X-Associated Neuropsychiatric Disorders (FXAND; Hagerman et al., 2018; Flavell et al., 2023). FXAND is highly prevalent and typically manifests earlier in life than other *FMR1*-related conditions, often during adolescence and early adulthood (Cordeiro et al., 2015; Wheeler et al., 2017). While some researchers have posited that this emotional dysregulation may serve as a prodromal phase for FXTAS in certain carriers (Hagerman et al., 2018), it is crucial to note that FXAND is not merely a prodrome. Unlike FXAND, which is prevalent among PMCs, the risk and clinical presentation of FXTAS differ significantly by sex. Females are at a substantially lower risk for developing FXTAS compared with males, and those affected typically manifest more subtle symptoms (Hagerman et al., 2004; Wheeler et al., 2017). Given that our study sample consists exclusively of female PMCs, FXTAS impacts this demographic much less frequently and severely. Also, because the underlying mechanisms of FXAND deviate from those driving neuropsychiatric conditions unrelated to the *FMR1* premutation, there is a distinct need for novel, targeted therapeutic interventions (Flavell et al., 2023). These findings highlight the urgent need to investigate the neural mechanisms underlying emotion dysregulation in female PMCs, to inform early detection and targeted intervention strategies.

Prior studies have investigated executive dysfunction in PMCs through neuroimaging approaches (Shelton et al., 2016; Moser et al., 2021). For instance, hyperactivation in visual cortices during emotional tasks has been reported (Bradley et al., 2003). Yet, other studies in premutation populations have yielded mixed evidence regarding prefrontal engagement (Hessl et al., 2007; Maltman et al., 2021). In addition, accumulated evidence suggested that PMCs have abnormalities in emotion processing (Bradley et al., 2003; Hessl et al., 2007; Brown and Stanfield, 2015). Increased activation in the amygdala and prefrontal cortex has been reported in PMCs during neutral face processing (Hessl et al., 2007). In another emotion processing task, PMCs exhibited a significant decrease in limbic-related activation (Hessl et al., 2011). While these findings suggest that PMCs exhibit pronounced emotion processing abnormalities, it remains unknown whether emotional interference disrupts their cognitive control. Moreover, asymmetric frontal activity has previously been linked to variations in emotion processing (Allen et al., 2004; Stewart et al., 2010; Smith et al., 2017). Changes in frontal asymmetry have been reported in individuals with the full mutation (Norris et al., 2022). However, recent evidence in PMCs has been more nuanced and they identified reduced neural responsivity to auditory stimuli in clusters associated with elevated neuropsychiatric symptoms and executive dysfunction, yet they did not find evidence of laterality in these responses (Norris et al., 2023). However, no studies have examined whether PMCs exhibit atypical lateralization patterns during emotion-directed cognitive tasks. Taken together, current evidence is insufficient to account for PMCs' integration of emotional processing, hemispheric coordination, and executive control.

To bridge these gaps, the present study aimed to investigate the neural response associated with the inhibitory control and conflict processing of PMCs. Hemodynamic response of the PMCs during an emotion-directed Stroop and Go/No-Go tasks was collected by a fNIRS system. By contrasting their brain activation to those of the control group, we aimed to elucidate the neural mechanism underlying the emotional processing and cognitive control of female PMCs, which might inform effective interventions for this clinically underrepresented population.

## Materials and Methods

### Participants

A total of 31 participants of Chinese descent were recruited at a location which will be identified if the article is published. Participants were divided into two groups: 14 participants (all females) with the *FMR1* premutation (PMCs group) and 17 healthy adult participants (3 male and 14 females, Control group).

Inclusion criteria for PMCs group: confirmed *FMR1* premutation allele (55–200 CGG repeats). Inclusion criteria for control group: no *FMR1* premutation or full mutation. Exclusion criteria for control group: neurodevelopmental or genetic disorders, bipolar disorder, psychosis, severe obsessive-compulsive disorder, Tourette's syndrome, birth injury, head trauma, or acute/severe infectious illnesses (e.g., encephalitis, meningitis, systemic infection with fever or marked malaise) that could transiently affect cognitive task performance, attention, or emotional state.

The intelligence quotients of PMCs were estimated by the Wechsler Adult Intelligence Scale (WAIS), Chinese version (Cui et al., 2017). The Symptom Checklist-90 (SCL-90) was administered to assess PMCs' multidimensional psychopathological symptoms using its 90 items (Wang, 1984), which comprise nine primary clinical subscales (somatization, obsessive-compulsive symptoms, interpersonal sensitivity, depression, anxiety, anger-hostility, phobic anxiety, paranoid ideation, and psychoticism) and seven supplementary items assessing additional symptoms such as sleep disturbances and appetite changes. Higher scores represent greater symptom severity across all domains. Concurrently, the Behavior Rating Inventory of Executive Function-Adult Version (BRIEF-A), a standardized 75-item self-report measure (Trujillo et al., 2020), was utilized to systematically evaluate PMCs' executive functioning through its Global Executive Composite (GEC) index, which comprises two higher-order dimensions: the Behavioral Regulation Index (including Inhibit, Shift, Emotional Control, and Self-Monitor subscales) and the Metacognition Index (including Initiate, Working Memory, Plan/Organize, Task Monitor, and Organization of Materials subscales). Higher scores indicate greater impairment in executive functioning. Written consent was obtained from each participant before the study, and all the procedures were performed following the Declaration of Helsinki.

### Functional NIRS data acquisition

**Figure 1.**
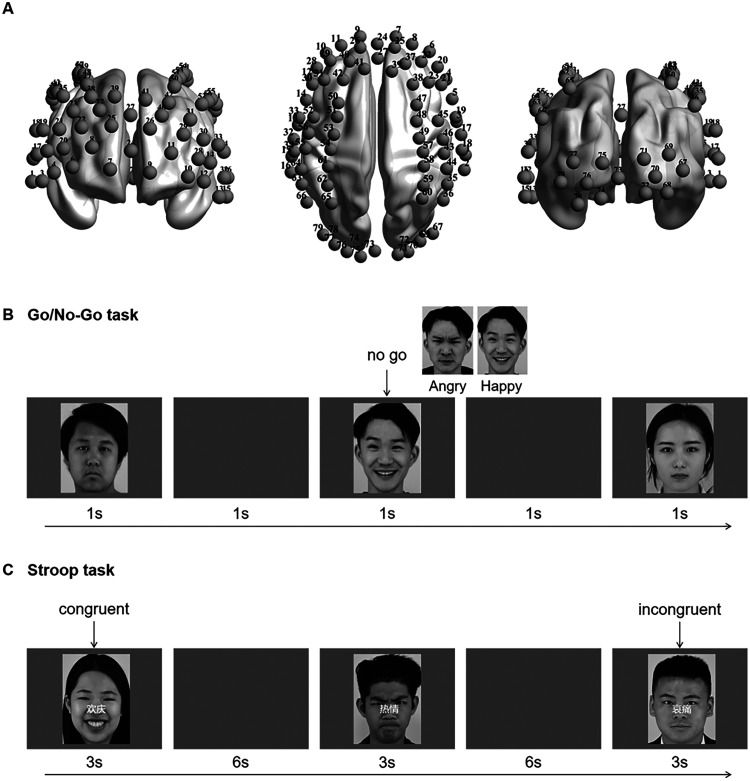
Overview of the experimental design. ***A***, The layout of fNIRS channels. ***B***, Experimental paradigm of the Go/No-Go task. ***C***, Experimental paradigm of the Stroop task.

Hemodynamic activity of the participants was recorded using a 79-channel continuous-wave fNIRS system (Danyang Huichuang Medical Equipment) operating at 730 and 850 nm wavelengths (11 Hz sampling rate). A customized cap containing 29 light sources and 30 light detectors was arranged and placed on the head of each participant. A total of 79 channels were divided by Brodmann area based on device coordinates, allowing the specified regions of interest (ROIs) to be delineated in this study. The layout of channels is illustrated in Figure 1*A*.

### Experiment paradigm

#### Go/No-Go task

Participants completed an emotional inhibition Go/No-Go task with two counterbalanced blocks (Happy and Angry No-Go blocks; Fig. 1*B*). The facial stimuli were selected from the Tsinghua facial expression database (Yang et al., 2020), which contains validated photographs of Chinese faces displaying various emotional expressions. All selected images were converted to grayscale and standardized for size (400 × 600 pixels) and luminance. In each block, participants were presented with 100 face stimuli, including 80 neutral faces (Go trials) and 20 emotional faces (No-Go trials) presented in a pseudorandomized order. The pseudorandomization ensured that no more than two No-Go trials occurred consecutively. Each trial began with the presentation of a face stimulus for 1,000 ms, followed by an intertrial interval (ITI) of 1,000 ms displaying a blank screen. The fixed interstimulus interval of 1,000 ms was employed to establish a rhythmic, prepotent motor response, thereby maximizing the inhibitory demand during No-Go trials. For Go trials (neutral faces), participants were instructed to respond as quickly as possible by pressing “0” on a numeric keypad. Two types of emotional faces, happy and angry, were used in each block of the No-Go trials, respectively. The participants were instructed to withhold their response in the No-Go trials. In the Happy block, the No-Go stimuli consisted of faces displaying happiness, while the No-Go stimuli consisted of faces displaying anger in the Angry block. A 1 min resting period was set between the two experimental blocks. The entire task was ∼8 min.

#### Stroop task

Participants completed an emotional Stroop task with the experimental process shown in Figure 1*C*. We selected 30 facial images (10 happy, 10 angry, and 10 neutral) from the Tsinghua facial expression database (Yang et al., 2020), which contains validated photographs of Chinese faces displaying various emotional expressions. All selected images were converted to grayscale and standardized for size (400 × 600 pixels) and luminance. Twenty emotional words were selected, including 10 positive words and 10 negative words. The three types of facial expressions and two types of words were paired to create six stimulus conditions: “happy face-positive word,” “happy face-negative word,” “angry face-positive word,” “angry face-negative word,” “neutral face-positive word,” and “neutral face-negative word.” The emotional word was superimposed on the center of the emotional face.

During the Stroop task, participants were instructed to identify whether the word represented a positive or negative emotion by pressing designated keys on the numeric keypad (“1” for positive, “2” for negative) as quickly and accurately as possible while ignoring the facial expression. A face–word compound stimulus appeared for 3,000 ms. The intertrial interval was randomly jittered between 5,500 and 6,500 ms. The experiment consisted of 60 trials in total, with each of the 6 stimulus conditions presented 10 times. No face or word was repeated across trials. The presentation order was pseudorandomized with the constraint that the same condition could not appear more than twice consecutively. The task was ∼10 min to complete.

### Data preprocessing

fNIRS data processing was using the NIRS AnalyzIR toolbox in MATLAB R2021a (Santosa et al., 2018). The raw fNIRS data were first converted to optical density. Motion artifacts were removed from the optical density using a Wavelet-based approach (Molavi and Dumont, 2012) and the Temporal Derivative Distribution Repair (TDDR) approach (Fishburn et al., 2019). A bandpass filter (0.01–0.5 Hz) was applied to remove the slow drift induced by physiological and environmental noise. Signal quality was conducted by wavelet transforms and visual inspection, and data with >50% of the total number of bad channels were excluded. The concentration changes of oxygenated hemoglobin (oxy-Hb) and deoxygenated hemoglobin (deoxy-Hb) were then computed according to the modified Beer–Lambert law. Oxy-Hb was selected as the primary indicator for this study due to its higher sensitivity (Strangman et al., 2002; Hoshi, 2003) and better signal-to-noise ratio than deoxy-Hb (Strangman et al., 2002).

### Statistical analysis

After the preprocessing of the raw fNIRS data, we performed first-level general linear model (GLM) analysis to identify brain activation in response to different conditions in each task for each participant. The canonical hemodynamic response function was used to construct the design matrix. The group-level analysis was performed based on the beta values of individual channels obtained from the first-level GLM to identify group-wise activation patterns using a linear mixed-effects model (LMM) implemented in the NIRS-Toolbox. The model was specified with the formula, where group and condition were treated as fixed effects, and participants were treated as random variables (Santosa et al., 2018):

formula="beta∼−1+group:cond+(1|age)".

To account for demographic differences, age was included as a nuisance covariate in the group-level analysis. Finally, distinct cortical activation between conditions and between groups was assessed using linear contrasts (General Linear Hypothesis Tests) within the LMM framework.

For behavioral analysis, independent *t* tests were used to test the differences between two groups in terms of age, task accuracy, and response time. We also used Kendall's correlation analysis to assess whether there was a significant correlation between brain activation and behavioral measures. All statistical analyses for behavioral data were conducted using MATLAB R2021a. Multiple comparison was corrected by controlling the false discovery rate (FDR) with the Benjamini–Hochberg procedure (*p* < 0.05).

### Sensitivity analysis for the female-only sample

To verify that the slight sex imbalance in the primary control group did not bias our main findings, a sensitivity analysis was performed. We reanalyzed the task-evoked brain activation by excluding the three male controls, resulting in a strictly female-matched subsample (14 PMCs vs 14 Controls). This sensitivity analysis focused on regional brain activation patterns.

## Result

### Demographic characteristics

Table 1 shows the statistical description of the clinical characteristics (i.e., WAIS, SCL-90, BRIEF-A) of the PMCs group and cognitive-behavioral assessments (i.e., task accuracy and reaction time) of the participants. For the PMCs group (all females, 36.93 ± 8.06 years; range, 26–60 years), a total of 11 participants from the Go/No-Go task and 12 from the Stroop task were included in the analysis after data quality inspection. For the Control group (3 male and 14 females, 30.86 ± 5.91 years; range, 23–42 years), a total of 16 participants from the Go/No-Go task and 17 from the Stroop task were included in the analysis after data quality inspection. For both tasks, the reaction time of the PMCs group was significantly longer than that of the control group, while there was no significant difference in the accuracy between the two groups (Table 1).

**Table 1.** The clinical characteristics and cognitive-behavioral measures of the participants

	PMC	Control	Statistics	
*n*	14	17		
Gender (F:M)	14:0	14:3		
			*t*	*p*
Age	36.93 ± 8.06	30.86 ± 5.91	2.27	0.03
WAIS				
Verbal Intelligence Quotient (VIQ)	102.18 ± 11.64	–	–	–
Performance Intelligence Quotient (PIQ)	105.36 ± 13.06	–	–	–
Full Scale Intelligence Quotient (FSIQ)	104.55 ± 11.92	–	–	–
SCL-90				
Somatization	14.43 ± 4.34	–	–	–
Obsessive-compulsive	14.64 ± 5.29	–	–	–
Interpersonal sensibility	9.93 ± 1.33	–	–	–
Depression	16.86 ± 4.47	–	–	–
Anxiety	11.71 ± 1.82	–	–	–
Anger-hostility	7.36 ± 1.65	–	–	–
Phobic anxiety	7.14 ± 0.36	–	–	–
Paranoid ideation	6.64 ± 1.01	–	–	–
Psychoticism	11.21 ± 1.89	–	–	–
Additional items	8.43 ± 2.41	–	–	–
BRIEF-A				
Behavioral Regulation Index (BRI)^a^	48.69 ± 10.05	–	–	–
Metacognition Index (MI)^a^	47.69 ± 10.14	–	–	–
Global Executive Composite (GEC)^a^	47.92 ± 10.66	–	–	
Go/No-Go				
Accuracy	0.98 ± 0.02	0.99 ± 0.01	−1.89	0.08
Reaction time	0.58 ± 0.07	0.50 ± 0.07	3.02	0.01
Stroop				
Accuracy (%)	0.95 ± 0.02	0.96 ± 0.01	−0.74	0.46
Reaction time (s)	1.08 ± 0.22	0.86 ± 0.16	2.94	0.01

“–” means the data is not available.

a Means *T* scores.

### Brain activation in response to the Go/No-Go task

**Figure 2.**
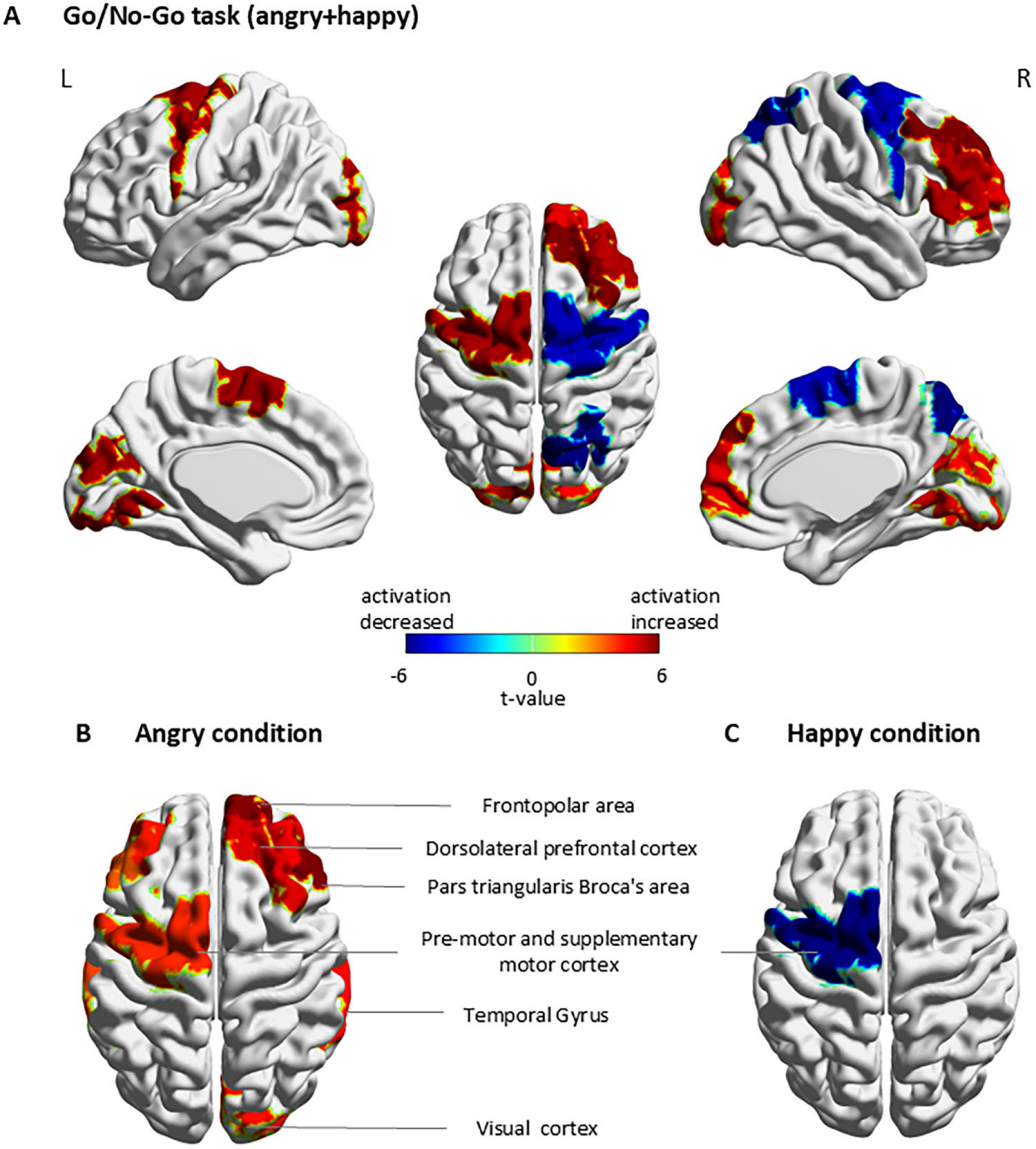
Comparison of brain activation in response to the Go/No-Go task between the PMCs and control groups. ***A***, Comparison of all blocks. ***B***, Comparison of the “angry” No-Go trials. ***C***, Comparison of the “happy” No-Go trials. Extended Data Figure 2-1 provides the complete brain activation for the female-only sample.

In the Go/No-Go task, significant differences in brain activation were observed across multiple ROIs between the PMCs and control groups (Fig. 2*A*). In prefrontal regions, PMCs exhibited increased activation in right-lateralized executive networks, including the right frontopolar area (FPC, *t* = 3.049, *q* = 0.026), right dorsolateral prefrontal cortex (DLPFC, *t* = 3.844, *q* = 0.014), and right pars triangularis of Broca's area (*t* = 3.456, *q* = 0.013). Hemispheric asymmetry of motor-related areas was identified in the PMCs, with left-lateralized increased activation in the premotor and supplementary motor area (SMA, left: *t* = 3.050, *q* = 0.026) contrasting with decreased activation in the right hemisphere (*t* = −3.260, *q* = 0.017). In addition, suppressed activity in the right somatosensory association cortex (*t* = −3.746, *q* = 0.012) and increased activation in the visual cortex (left: *t* = 3.504, *q* = 0.013; right: *t* = 2.854, *q* = 0.041) were also identified. The significantly activated ROIs are listed in Table 2.

10.1523/ENEURO.0031-26.2026.f2-1 Figure 2-1 Comparison of brain activation in response to the Go/No-Go task between the female-only sample. (A) Comparison of all blocks; (B) Comparison of the ‘angry’ No-Go trials; (C) Comparison of the ‘happy’ No-Go trials Download Figure 2-1, TIF file.

**Table 2.** Summary of the statistically activated ROIs during the Go/No-Go task

	Channel	Brodmann area	Beta	*t*	*p*	*q*-FDR	Power	Cohen's *d*
All	6	10—frontopolar area	45.253	3.049	0.003	0.026	0.706	1.224
	20	46—dorsolateral prefrontal cortex	39.890	3.359	0.001	0.014	0.802	1.349
	21	45—pars triangularis Broca's area	41.465	3.456	0.001	0.013	0.828	1.388
	23	9—dorsolateral prefrontal cortex	46.935	3.844	<0.001	0.012	0.908	1.544
	33	6—premotor and supplementary motor cortex	63.975	3.539	0.001	0.013	0.848	1.421
	48	6—premotor and supplementary motor cortex	−80.420	−3.260	0.002	0.017	0.774	−1.309
	52	6–premotor and supplementary motor cortex	65.209	3.050	0.003	0.026	0.706	1.225
	60	7—somatosensory association cortex	−95.312	−3.746	<0.001	0.012	0.891	−1.504
	72	18—visual association cortex (V2)	87.147	2.854	0.005	0.041	0.636	1.146
	74	18—visual association cortex (V2)	116.452	3.504	0.001	0.013	0.840	1.407
Angry	1	21—middle temporal gyrus	55.114	3.536	0.001	0.005	0.848	1.420
	4	45—pars triangularis Broca's area	49.109	4.870	<0.001	<0.001	0.990	1.955
	6	10—frontopolar area	49.463	4.782	<0.001	<0.001	0.988	1.920
	7	10—frontopolar area	26.728	3.879	<0.001	0.002	0.914	1.558
	17	22—superior temporal gyrus	55.062	3.626	<0.001	0.004	0.867	1.456
	20	46—dorsolateral prefrontal cortex	48.516	5.873	<0.001	<0.001	0.999	2.358
	21	45—pars triangularis Broca's area	35.890	4.317	<0.001	0.001	0.963	1.733
	22	46—dorsolateral prefrontal cortex	27.586	3.663	<0.001	0.004	0.875	1.471
	23	9—dorsolateral prefrontal cortex	33.322	3.947	<0.001	0.002	0.924	1.585
	29	46—dorsolateral prefrontal cortex	27.033	3.452	0.001	0.006	0.827	1.386
	30	45—pars triangularis Broca's area	26.416	2.794	0.006	0.035	0.613	1.122
	33	6—premotor and supplementary motor cortex	41.199	3.280	0.001	0.009	0.780	1.317
	34	22—superior temporal gyrus	38.873	2.997	0.003	0.021	0.688	1.204
	70	17—primary visual cortex (V1)	55.029	2.751	0.007	0.037	0.597	1.105
	72	18—visual association cortex (V2)	74.803	3.531	0.001	0.005	0.846	1.418
Happy	51	6—premotor and supplementary motor cortex	−42.917	−2.493	<0.001	0.032	0.495	−1.468

10.1523/ENEURO.0031-26.2026.t2-1 Table 2-1 All result of the Go/No-Go task (for PMC:Controls = 14:17). Download Table 2-1, XLS file.

10.1523/ENEURO.0031-26.2026.t2-3 Table 2-3 All result of the Go/No-Go task (for female-only). Download Table 2-3, XLS file.

We further examined the brain activation in response to different emotional face stimuli in the No-Go condition. In the “angry” No-Go condition, PMCs exhibited a right-lateralized activation pattern within frontal and temporal cognitive control networks (Fig. 2*B*), including increased activation in the right FPC (*t* = 4.782, *q* < 0.001), right DLPFC (*t* = 5.873, *q* < 0.001), right middle temporal gyrus (*t* = 3.536, *q* = 0.005), and right superior temporal gyrus (*t* = 3.626, *q* = 0.004). Increased activation was also identified in left-lateralized language and motor-related areas, specifically Broca's area (*t* = 4.317, *q* = 0.001), and the left SMA (*t* = 3.280, *q* = 0.009). Additionally, in the “happy” No-Go condition (Fig. 2*C*), the PMCs group only exhibited increased activation in the left SMA (*t* = −2.493, *q* = 0.032).

### Brain activation in response to the Stroop task

**Figure 3.**
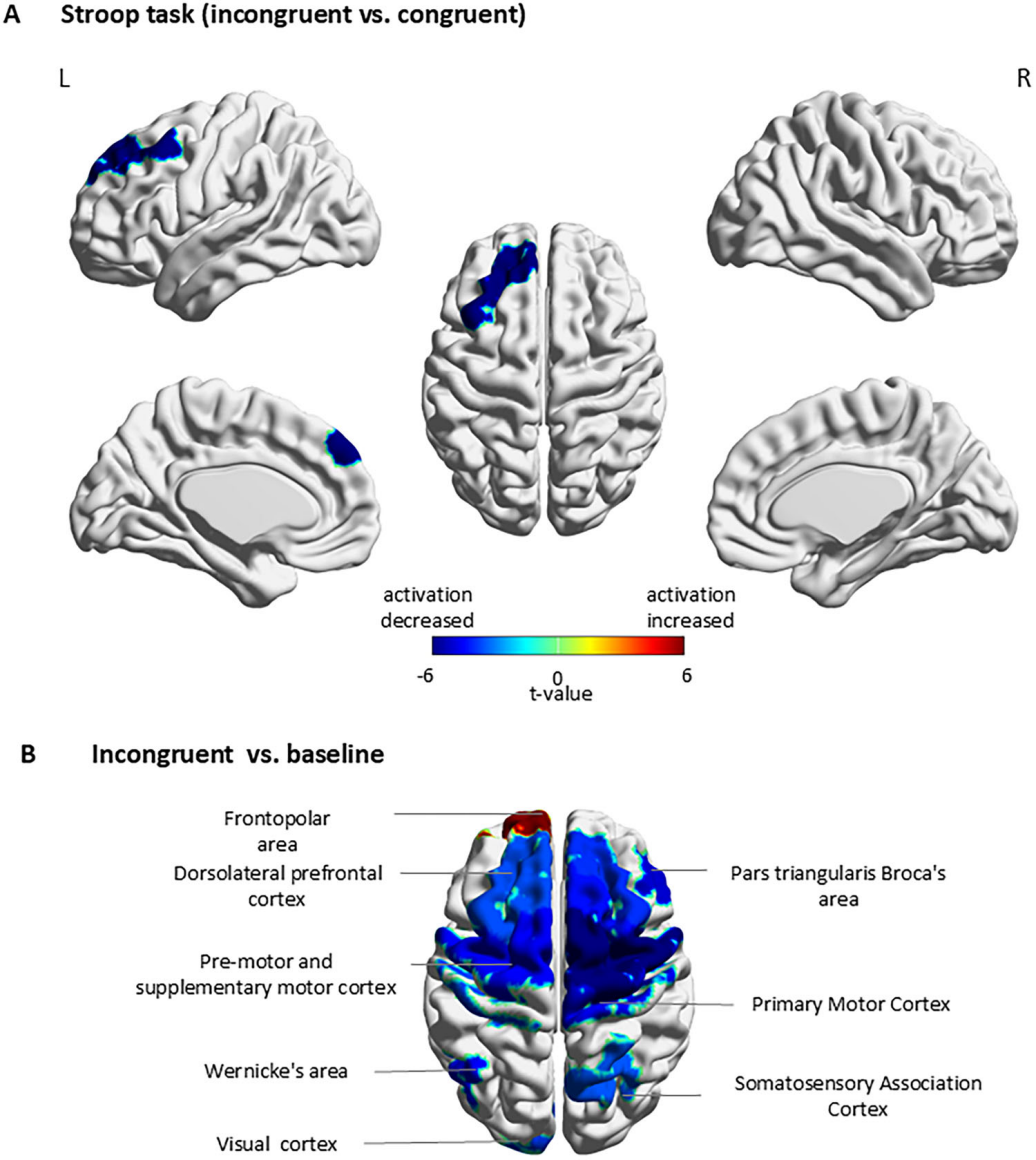
Comparison of brain activation in response to the Stroop task between the PMCs and control groups. ***A***, Comparison of brain activation for contrast between incongruent and congruent conditions. ***B***, Comparison of brain activation for contrast between incongruent and baseline. Extended Data Figure 3-1 provides the complete brain activation for the female-only sample.

In the Stroop task, significant between-group differences of task-evoked activation (incongruent vs congruent) were identified in only one ROI between the PMCs and control groups (Fig. 3*A*). The PMCs group exhibited decreased activation in left DLPFC (*t* = −3.642, *q* = 0.029), as summarized in Table 3.

10.1523/ENEURO.0031-26.2026.f3-1 Figure 3-1 Comparison of brain activation for contrast between incongruent and baseline in the female-only sample during the Stroop task Download Figure 3-1, TIF file.

**Table 3.** Summary of the statistically activated ROIs during the Stroop task (cong, congruent; incong, incongruent)

	Channel	Brodmann area	Beta	*t*	*p*	*q*-FDR	Power	Cohen's*d*
Incong vs cong	40	9—dorsolateral prefrontal cortex	−3.302	−3.642	<0.001	0.029	0.873	−1.405
Incong vs baseline	21	45—pars triangularis Broca's area	−4.039	−3.860	<0.001	0.006	0.913	−1.489
	23	9—dorsolateral prefrontal cortex	−2.840	−3.024	0.003	0.029	0.700	−1.167
	26	10—frontopolar area	2.705	2.805	0.006	0.040	0.621	1.082
	41	9—dorsolateral prefrontal cortex	−2.597	−2.754	0.007	0.040	0.601	−1.062
	45	6—premotor and supplementary motor cortex	−7.936	−4.582	<0.001	0.001	0.981	−1.768
	47	8—includes frontal eye fields	−4.311	−3.341	0.001	0.014	0.800	−1.289
	48	6—premotor and supplementary motor cortex	−4.519	−2.674	0.008	0.043	0.570	−1.031
	50	8—includes frontal eye fields	−5.219	−2.756	0.007	0.040	0.601	−1.063
	53	6—premotor and supplementary motor cortex	−7.176	−3.491	0.001	0.011	0.839	−1.347
	55	3—primary somatosensory cortex	−4.361	−2.806	0.006	0.040	0.621	−1.083
	57	4—primary motor cortex	−5.303	−3.072	0.002	0.028	0.717	−1.185
	58	4—primary motor cortex	−7.245	−3.573	<0.001	0.011	0.858	−1.378
	59	7—somatosensory association cortex	−4.922	−2.680	0.008	0.043	0.572	−1.034
	66	39—angular gyrus–part of Wernicke’s area	−4.737	−3.455	0.001	0.011	0.830	−1.333
	75	17—primary visual cortex (V1)	−7.448	−2.909	0.004	0.036	0.659	−1.122

10.1523/ENEURO.0031-26.2026.t3-1 Table 3-1 All result of the Stroop task (for PMC:Controls = 14:17). Download Table 3-1, XLS file.

10.1523/ENEURO.0031-26.2026.t3-3 Table 3-3 All result of the Stroop task (for female-only). Download Table 3-3, XLS file.

Contrasting incongruent with baseline conditions revealed distinct activation patterns in the PMCs group relative to controls (Fig. 3*B*). The PMCs group exhibited significantly increased activation in the left FPC (*t* = 2.805, *q* = 0.040). Conversely, decreased activation was observed bilaterally in the DLPFC (right *t* = −3.024, *q* = 0.029; left *t* = −2.754, *q* = 0.040) and SMA (right *t* = −4.582, *q* = 0.001; left *t* = −3.491, *q* = 0.011). Unilateral decreases were identified in the right primary motor cortex (*t* = −3.573, *q* = 0.011), right somatosensory association cortex (*t* = −2.680, *q* = 0.043), and left primary somatosensory cortex (*t* = −2.806, *q* = 0.040). Language-related regions showed suppressed activation, including the left Wernicke's area (*t* = −3.455, *q* = 0.011) and right pars triangularis of Broca's area (*t* = −3.860, *q* = 0.006).

### Brain–behavior association analysis

**Figure 4.**
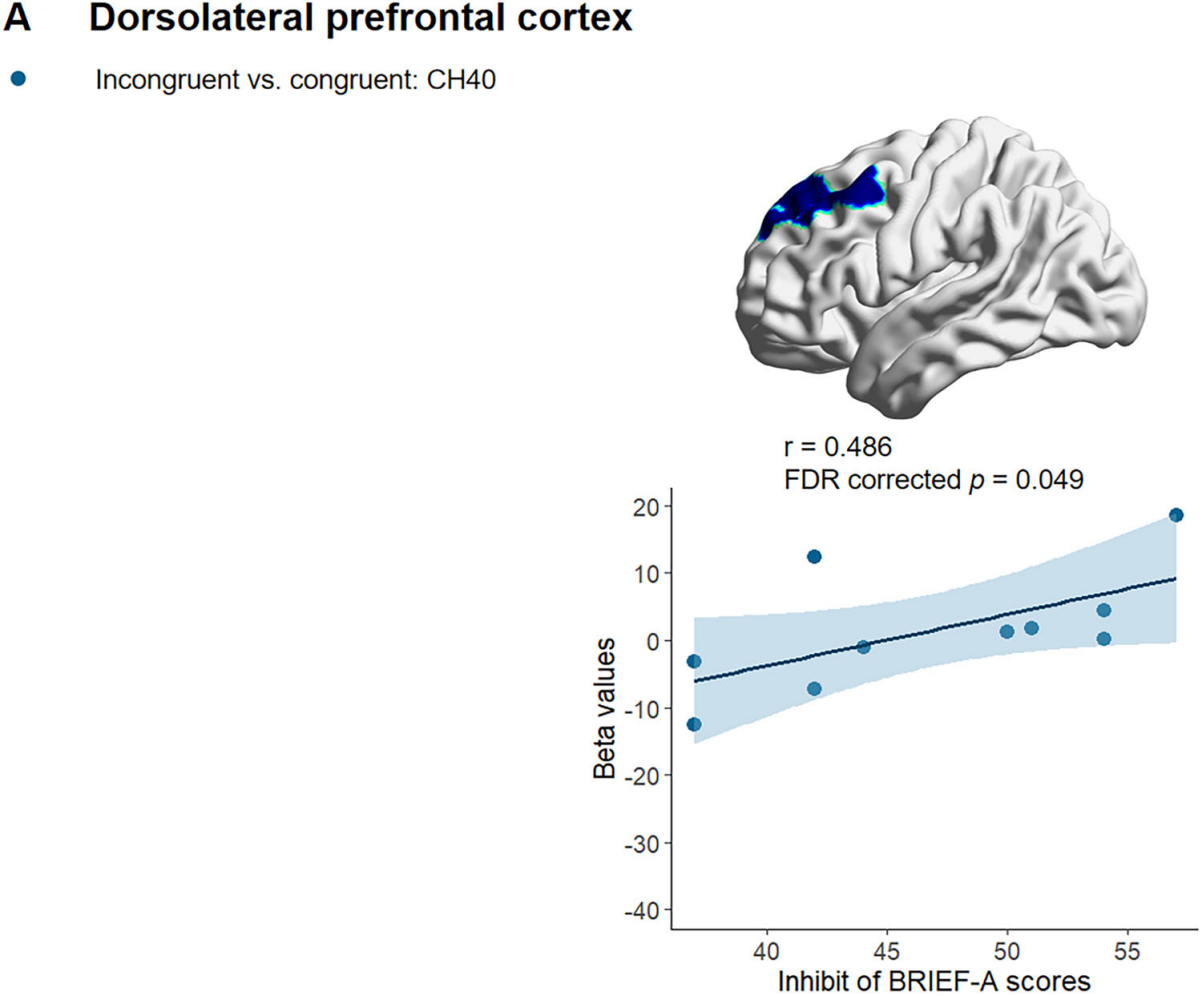
The correlation between the brain activation in the dorsolateral prefrontal cortex during the Stroop task (incongruent vs congruent) and the inhibition subscale of BRIEF-A; Extended Data Figure 4-1 provides the all results of the Kendall's correlation analysis across all channels (including nonsignificant correlations).

The Kendall's correlation analysis found that the inhibition ability, measured by the BRIEF-A, was significantly positively correlated with the brain activation in the left DLPFC (*r* = 0.486, *p* = 0.049) during the Stroop task (incongruent vs congruent). That is, greater impairment of the executive function of PMCs was associated with greater brain activation in left DLPFC (Fig. 4; see Extended Data Fig. 4-1 for all results of the brain–behavior association analysis).

10.1523/ENEURO.0031-26.2026.f4-1 Figure 4-1 All result of Brain-behavior association analysis. Download Figure 4-1, XLS file.

### Sensitivity analysis for the female-only sample

To ensure the robustness of our brain activation findings against potential sex effects, we evaluated the female-only subsample (14 PMCs vs 14 Controls). For the Go/No-Go task, the reanalysis largely recapitulated the primary findings. Overall, PMCs exhibited a consistent pattern of increased activation in right-lateralized executive networks, including the right frontopolar area (FPC; *t* = 3.341, *q* = 0.044) and right dorsolateral prefrontal cortex (DLPFC; *t* = 3.240, *q* = 0.044), alongside suppressed activity in the right somatosensory association cortex (*t* = −4.969, *q* < 0.001; Extended Data Fig. 2-1*A*). Under specific emotional valences, PMCs showed right-lateralized frontal-temporal hyperactivation during the “angry” No-Go condition (e.g., right FPC: *t* = 5.373, *q* < 0.001; right DLPFC: *t* = 5.254, *q* < 0.001; Extended Data Fig. 2-1*B*). Conversely, a predominantly left-lateralized hypoactivation across frontal, parietal, and temporal regions was observed during the “happy” No-Go condition (e.g., left premotor/SMA: *t* = −4.053, *q* = 0.004; left DLPFC: *t* = −3.621, *q* = 0.008; Extended Data Fig. 2-1*C*). The comprehensive channel-wise statistical outputs and activation maps for this task are detailed in Extended Data Table 2-2 and Figure 2-1, respectively.

10.1523/ENEURO.0031-26.2026.t2-2 Table 2-2 Summary of the statistically activated ROIs during the Go/No-Go task(for female-only). Download Table 2-2, XLS file.

For the Stroop task, consistent with the primary cohort, no significant group differences in task-evoked activation were identified for the primary contrast (incongruent vs congruent). However, the contrast between incongruent and baseline conditions revealed significantly increased activation in the left FPC (*t* = 3.238, *q* = 0.012) and extensive decreases within bilateral motor and somatosensory networks in PMCs relative to controls (e.g., right primary motor cortex: *t* = −4.637, *q* = 0.001; left premotor/SMA: *t* = −4.161, *q* = 0.001; Extended Data Fig. 3-1). These regional activation data and corresponding brain maps are compiled in Extended Data Table 3-2 and Figure 3-1.

10.1523/ENEURO.0031-26.2026.t3-2 Table 3-2 Summary of the statistically activated ROIs during the Stroop task (for female-only). Download Table 3-2, XLS file.

## Discussion

Cognitive-behavioral dysfunction in PMCs primarily involves deficits in executive function, inhibition, and visuospatial learning (Bourgeois et al., 2007), yet the neural mechanisms underlying these deficits remain unclear. The present study has uncovered distinct neural activation patterns in PMCs compared with healthy controls during cognitive control tasks (Go/No-Go and Stroop). Whole-brain GLM analyses revealed significant between-group differences across multiple brain regions in response to emotion-directed cognitive processing. These findings complement previous reports of neuropsychiatric and cognitive phenotypes in PMCs (Shelton et al., 2017; Moser et al., 2021), advancing our current understanding of the cognitive executive deficits and emotion-specific processing abnormalities in PMCs.

We found that PMCs exhibited increased activation in the DLPFC during the Go/No-Go task. The increased DLPFC activity, together with the longer reaction time, may reflect greater cognitive effort to sustain task accuracy. The overactivation in PMCs likely reflects impaired synaptic efficiency, driven by *FMR1* mRNA toxicity and partial loss of FMRP function. Elevated levels of expanded CGG-repeat *FMR1* mRNA may sequester RNA-binding proteins, disrupt RNA metabolism, and trigger cellular stress responses, ultimately compromising synaptic homeostasis (Tassone et al., 2000; Hagerman et al., 2011; Hessl et al., 2011). In addition, decreased brain activity in the premotor and SMA during the Go/No-Go task was identified in the PMCs, possibly reflecting weaker cognitive control and motor coordination. Previous studies have reported that PMCs are at risk for age-related neurodegeneration that may result in motor and/or cognitive impairments (Hagerman et al., 2001, 2004). Although the individuals in the current sample do not present with clinical symptoms of FXTAS, these observed neural disruptions may represent subthreshold features inherent to all carriers or serve as early neurobiological markers predictive of future clinical decline.

PMCs exhibited decreased activation in the DLPFC during the Stroop task, which is contrary to the result of the Go/No-Go task. This task-dependent dissociation aligns with the compensatory overactivation and resource depletion. That is, the Go/No-Go task predominantly engages proactive inhibition, requiring DLPFC-driven effortful control (Rubia et al., 2001), whereas the Stroop task relies on reactive conflict resolution involving dynamic interactions between DLPFC and language networks (MacDonald et al., 2000; Freund et al., 2021). These task-dependent deficits in executive processes may be attributed to the neurobiological effects of *FMR1* mRNA toxicity. While not directly tested in the current study, it is hypothesized that elevated *FMR1* mRNA levels and subsequent neuronal dysfunction potentially compromise synaptic plasticity and neural efficiency within the prefrontal cortex, thereby contributing to the atypical activation patterns observed in PMCs.

Our brain activation–behavior association analysis found that the inhibition ability of the PMCs was significantly positively correlated with brain activation of the DLPFC during the Stroop task, indicating that worse inhibitory executive function of the PMCs could be attributed to hypoactivation of the DLPFC. The DLPFC is primarily believed to regulate emotional processing in a top-down manner through its inhibitory connections to other brain regions, such as the anterior cingulate cortex and amygdala (Drevets and Raichle, 1992; Davidson, 2002; Banks et al., 2007). The significant brain–behavior association found in our study suggests that neural response in DLPFC may be a sensitive biomarker for assessing the emotion-specific inhibitory control in PMCs, informing the development of targeted interventions in further clinical trial.

We found that PMCs revealed distinct brain activation patterns in response to different emotional stimuli in the tasks. For instance, the angry condition of the Go/No-Go task induced broader brain activation, such as the prefrontal cortex and right temporal lobe, which differs significantly from the activation induced by the happy condition. These atypical neural activation patterns suggest the potential dysfunction of emotion processing in PMCs. Previous structural MRI studies have reported reduced amygdala volume in PMCs (Hessl et al., 2011), and they also showed reduced activation in the right amygdala during an emotional processing task (Hessl et al., 2007). While fNIRS cannot directly measure activity of subcortical regions (e.g., amygdala) due to depth limitation, our finding here may reflect the abnormality of the prefrontal–amygdala loop in PMCs, a critical neural loop involved in regulating emotions (Gold et al., 2016; Delli-Pizzi et al., 2017). The present study suggests that future investigation of emotional dysfunction may help to unravel the neurodevelopmental aspects of the PMCs.

Another key finding is the hemispheric asymmetry in the task-evoked brain activation. When engaging in the Go/No-Go tasks, the PMCs group revealed increased activation in the right FPA and DLPFC but not in the left hemisphere. Conversely, the PMCs group displayed increased activation in the left DLPFC during the Stroop task, but not in the opposite hemisphere. These frontal asymmetries resemble patterns that have been observed in depression (Smith et al., 2017; Norris et al., 2022, 2023; Tseng et al., 2022), which may arise from CGG repeat-related RNA toxicity (Hagerman et al., 2018; Hoem et al., n.d.). While premutation-associated RNA aggregates could independently impair interhemispheric communication through mitochondrial dysfunction or aberrant dendritic translation (Giovedí et al., 2014; Wang et al., 2017). Dysregulation of this pathway may disrupt interhemispheric balance by impairing activity-dependent refinement of hemispheric specialization (Nakamoto et al., 2007; Hagerman et al., 2011). Our findings may therefore further support a role of altered brain lateralization in ASD, warranting further investigations in this direction.

Several limitations should be acknowledged in this study. First, the present study adopted a cross-sectional design; we cannot determine causality or whether the observed PMCs impairments reflect neurodevelopmental differences or neurodegenerative processes. Second, although the *FMR1* premutation is not extremely rare, with an estimated global prevalence of up to 1 in 300 that varies by region (Hantash et al., 2011; Seltzer et al., 2012), recruiting these individuals presents a significant screening challenge. Because premutation carriers generally present as typically developing, they are largely identified only after a primary relative is diagnosed with the *FMR1* full mutation. Consequently, our research sample size is constrained by these screening difficulties and may be biased by the prevalence rate and ascertainment of fragile X syndrome. Future studies with larger, rigorously age-matched cohorts are warranted to fully understand the dynamics and origins of cognitive conflict processing deficits in PMCs. In addition, fNIRS measures cortical hemodynamics but has limited penetration depth. Thus, it is unclear whether there was a difference in deeper brain structures between PMCs and controls. Furthermore, the precise localization of the fNIRS channels has to be considered with caution. Future studies could use whole-brain imaging techniques such as MRI to validate and expand the findings in our study. Also, all participants in this study were of Chinese descent, and the facial stimuli utilized in our tasks were selected from a Chinese facial expression database. While matching the racial background of participants and stimuli eliminates the other-race effect confound, the well-documented own-race advantage may have facilitated emotional recognition and influenced response speeds in our tasks (Elfenbein and Ambady, 2002). Consequently, the generalizability of these findings to cross-cultural or diverse racial populations remains to be established.

### Conclusion

Our fNIRS study demonstrates distinct neural activation patterns in PMCs during emotion-driven inhibitory control (Go/No-Go) and conflict processing (Stroop) tasks compared with controls. Understanding these task-specific and regionally distinct compensatory mechanisms provides critical neurobiological insights into PMCs-related executive dysfunction. This foundation can inform the development of targeted interventions, such as neurostimulation or cognitive training strategies focused on these dysfunctional circuits. However, future large-scale longitudinal and multimodal studies remain essential to confirm causality and optimize clinical translation.

## Synthesis

Reviewing Editor: Alexander Soutschek, Universitatsklinikum Wurzburg Decisions are customarily a result of the Reviewing Editor and the peer reviewers coming together and discussing their recommendations until a consensus is reached. When revisions are invited, a fact-based synthesis statement explaining their decision and outlining what is needed to prepare a revision will be listed below. The following reviewer(s) agreed to reveal their identity: Jordan Norris.

The manuscript "Aberrant Emotion-Cognition Integration in FMR1 Premutation Carriers: a

fNIRS Study" details findings related to disruptions in cognitive processes in FMR1 PMCs under emotional load. This study adds a novel perspective on PMC neurophysiology that is important for the field. Disruptions to core areas related to inhibitory control and sensory integration highlights domains that will better inform how the FMR1 premutation impacts neurophysiology, as well as represent potential biomarkers that can be employed to support therapeutic efficacy in trials targeting symptoms of FXAND.

The manuscript is well written, and the revisions requested by the prior reviewer were thoughtfully addressed in a manner that increases the rigor of the study/manuscript. I find the manuscript is publishable in its current state, though it would be made stronger by addressing the following items:

1. On lines 59-68 the authors discuss FXAND and FXTAS followed by commentary on emotional dysregulation in female PMCs. While FXAND affects all PMCs, FXTAS does not (as acknowledged in the authors' comments to the previous review). I think it's important to add that nuance to lines 59-68 because FXAND is not just a prodromal phase for FXTAS, as women are largely protected from FXTAS. Especially because all PMCs in the study are female, it may also add study-specific nuance to note that FXTAS does not typically impact female PMCs.

2. Please provide race/ethnicity information on the participants, if available. The reason this is important is that the study tasks used stimuli selected from a database that only included Chinese faces. There are a number of studies that document differences in affective processing (identification of emotions) between images of individuals of the same race as the participant and images of those from other races than the participant (own-race advantage). As such, it's important to know the racial/ethnicity composition of the sample to fully consider the results. Please also note the effect of same-race/other-race emotional processing differences as a potential confounding variable on response speed, if applicable to the study.

3. Please document which statistical analysis software was used for all stats reported.

4. The limitations section acknowledges the rarity of FXS but not why PMC samples are limited. The FMR1 premutation has a higher prevalence than FXS, at roughly 1 in 200 in the US and up to 1 in 300 worldwide (varies by region), which does not qualify as rare. Rather, we have a screening problem for the premutation. I recommend the authors also acknowledge that PMCs present as typically developing and are largely identified because they are the primary relative of individuals with the FMR1 full mutation (FXS), so our research sample is biased by the prevalence rate of FXS.
